# Snow Mountain Virus recovery by synthetic human histo-blood group antigens is heavily influenced by matrix effects

**DOI:** 10.1038/s41598-020-60639-6

**Published:** 2020-03-13

**Authors:** Amy E. Kirby, Yvonne Kienast, Milagros Aldeco, Molly Steele, Abasaheb N. Dhawane, Dandan Liu, Xikai Cui, Amrita Das, Suri Iyer, Christine L. Moe

**Affiliations:** 1 Center for Global Safe Water, Sanitation and Hygiene, Rollins School of Public Health, Emory University, Atlanta, Georgia United States of America; 2 Center for Diagnostics and Therapeutics, Department of Chemistry, Georgia State University, Atlanta, Georgia United States of America; 3 Present Address: Division of Foodborne, Waterborne, and Environmental Diseases, Centers for Disease Control and Prevention, Atlanta, GA USA

**Keywords:** Microbiology, Virology, Carbohydrate chemistry

## Abstract

Noroviruses are known to bind to histo-blood group antigens (HBGAs) and the specific binding patterns depend on the virus genotype. However, the development of point-of-care diagnostic assays based on this binding has been challenging due to low assay sensitivity. This study utilized a well-defined stool collection from a GII.2 Snow Mountain Virus (SMV) human challenge study to investigate virus recovery from stool and emesis samples using HBGA-coated beads. SMV was recovered from H type III-coated beads for 13 stool specimens out of 27 SMV-positive specimens tested. After adjusting for non-specific binding to PEG-coated beads, the mean percent recovery by H type III-coated beads was 308.11% +/− 861.61. Recovery by H type III ligands was subject-specific and weakly correlated with stool consistency. Input virus titer was not correlated with SMV recovery. The results suggest that the generally low virus recovery we observed may be due to bead saturation or hindrance by existing glycans in the matrix that precluded the virus from being captured by the synthetic glycans. These results indicate a strong role for subject-specific and matrix effects in HBGA binding by SMV. Further investigation of the nature of this interference is needed to facilitate development of high sensitivity diagnostic assays.

## Introduction

Noroviruses are a major cause of gastroenteritis worldwide in all age groups^1^. In the United States, it is estimated to cause 58% of all foodborne illnesses^2^. The illness is generally limited and resolves without medical intervention, however, severe outcomes and deaths do occur^3^. Three norovirus genogroups are known to cause illness in humans: GI, GII, and GIV. GII viruses are the most common, specifically GII.4, which is the genotype responsible for cyclic norovirus pandemics^4^. *In vitro* studies have shown that noroviruses bind to histo-blood group antigens (HBGAs), which comprise the ABO, Lewis, and secretor phenotypes^5^. HBGAs are thought to be the primary receptors for the virus, and the pattern of binding to specific HBGAs varies by genotype.

In clinical practice, norovirus illness is generally diagnosed without a laboratory test due to the lack of clinically useful diagnostics^6^. The gold standard diagnostic is reverse transcription-polymerase chain reaction (RT-PCR), which detects the viral RNA. For accurate genotype identification, RT-PCR is followed by amplicon sequencing. While RT-PCR is sensitive and specific, the fairly long turnaround time limits its utility for an illness where duration is measured in hours. Other antibody-based diagnostics are available, but their sensitivities are much lower than RT-PCR. Currently, there exists an unmet need to develop point of care diagnostics to detect norovirus in a variety of settings as early detection and isolation of the infected person would decrease the spread of the virus.

Until recently, noroviruses could not be cultured in the laboratory^7^, which limited diagnostic development to the use of recombinant capsid protein that formed virus-like particles (VLPs) and two sources of human stool specimens: outbreaks and human challenge trials. Outbreak specimens are available for nearly all norovirus genotypes, but the quantity and volume of samples are limited, and little is known about the donor or course of infection. Conversely, samples from human challenge trials are available in larger volumes, are collected throughout the course of infection, and have a wealth of associated metadata. However, only a handful of strains have been used in these challenges^8^. Due to the limited number of viral genotypes tested, specimens from challenge studies are rarely used for diagnostic development, however, these specimens can provide a valuable sample set to assess subject-to-subject variability and matrix effects.

In this study, synthetic HBGAs were assessed for their capacity to recover native GII.2 Snow Mountain Virus (SMV) from stool samples from infected subjects in a human challenge study as a first step towards the development of glycan-based diagnostics. These synthetic carbohydrates have previously been shown to bind GI.1 Norwalk virus VLPs and native Norwalk virus^9^. SMV VLPs are known to bind to H-type I carbohydrates, but not other HBGAs, such has H-type III, A, B, or the Lewis antigens^5^. By investigating the recovery of native virus from well-characterized stool specimens, this study elucidates a variety of subject and matrix factors that can impact SMV recovery by glycan-coated beads.

## Materials and Methods

### Synthetic histo-blood group antigens

Histo-blood group antigens H-type I, H-type III, A, and B were synthesized with polyethylene glycol (PEG) linkers and biotin moieties for conjugation to streptavidin beads. The synthetic process was previously described in detail^9^. Biotinylated PEG and biotinylated galactose were also synthesized and used as negative controls.

### Stool and emesis samples from a norovirus human challenge trial

Archived stool and emesis samples from a previous Snow Mountain Virus challenge trial were used. The challenge protocol was previously described^10^ and was approved by the University of North Carolina, Chapel Hill Institutional Review Board. All stools and emesis produced during the five-day inpatient period following challenge were collected. Stools were also collected at days 7, 14, 21, and 35 post-challenge. Pre-challenge stool samples were collected from each subject. All stools were graded for consistency and frozen at −80 °C. This study examined a total of 32 stool and 4 emesis specimens from five infected subjects. The stool specimens included five pre-challenge specimens and 27 SMV-positive specimens (as determined by RT-qPCR) from days 1–17 post-challenge. We used archived human samples for this study without any of the investigators having any access to the sample identifiers. Therefore, the study is exempt according to 45 CFR 46.101 (b)4. “Research involving collection or study of existing data, documents, records, pathological specimens, or diagnostic specimens, if these sources are publicly available or if the information is recorded by the investigator in such a manner that subjects cannot be identified, directly or through identifiers linked to the subjects”.

### Sample preparation

Stool suspensions (20% vol/vol) and emesis suspensions (50% vol/vol) were prepared in molecular water (Corning, Manassas, VA). All suspensions were stored at 4 °C overnight. On the next day and prior to usage in the bead-binding assay, the suspensions were centrifuged for 10 minutes at maximum speed (15,974 × g). The clarified supernatants were used for the bead-binding assays and reverse transcription-quantitative polymerase chain reaction (RT-qPCR) to determine input virus concentrations.

### Bead-binding assays

For each sample, bead-binding assays were run in parallel for six biotinylated synthetic carbohydrate ligands: H type I, H type III, A antigen, B antigen, galactose, and polyethylene glycol (PEG). For each bead-binding assay, 50 µl Dynabead MyOne Streptavidin T1 (Invitrogen, Carlsbad, CA) were washed three times with 1 ml of phosphate buffered saline (PBS, Corning, Manassas, VA). The beads were vortexed after adding the PBS. After a final suspension in 50 µl of PBS, 50 µl of synthetic carbohydrates (1 mg/ml in PBS) was added. The beads were incubated for 2 hours at room temperature in an end-over-end mixer. The beads were washed once in PBS +0.25% Tween 80 (PBST) to remove unbound carbohydrates (Corning; RPI, Mount Prospect, IL). The beads were blocked in 1 ml 10% skim milk (Alpha Diagnostic International Inc., San Antonio, TX) in PBST with continuous gentle rotation overnight at 4 °C. The beads were washed three times in PBS and resuspended in 1 ml PBS. For the viral capture step, 10 µl of each centrifuged stool or emesis sample was added to the prepared beads and the reaction was incubated at room temperature for 4 hours in an end-over-end mixer. Unbound virus was removed by washing five times with 1 ml PBST, followed by three washes with 1 ml RNAse-free PBS (Corning). The beads were resuspended in 50 µl RNase-free PBS. Replicate assays were not performed for all samples due to limitations in the availability of the synthetic carbohydrate ligands.

### Virus quantification

Viral RNA was extracted from the input stool or emesis suspensions and the bead-bound virus using the heat release method^11^. For each assay, 3 µl of the final bead suspension was added to 26.4 µl of reagent-grade water (Corning) and 0.6 µl of RNasin Plus ribonuclease inhibitor (Promega, Madison, WI), heated to 99 °C for five minutes and chilled on ice for two minutes. Viral RNA was quantified using a previously described SMV-specific RT-qPCR performed in duplicate^12^. RNA genome equivalent copies (GEC) were estimated from a standard curve generated from *in-vitro* transcribed SMV RNA. Standard curves were performed in duplicate on each RT-qPCR assay plate.

### Data analysis

For each sample, the estimated RNA GEC recovered from the PEG-coated beads was subtracted from the estimated RNA GEC recovered from the beads with the other five ligands to adjust for non-specific binding. These PEG-adjusted values were used to calculate percent recovery for each carbohydrate ligand (bead GEC/input GEC × 100%). HBGA-specific recovery was defined as PEG-adjusted percent recovery at least three times greater than the PEG-adjusted percent recovery by galactose-coated beads. Statistical analysis was conducted in Prism 7 (GraphPad Software, La Jolla, CA).

## Results

**Figure 1:**
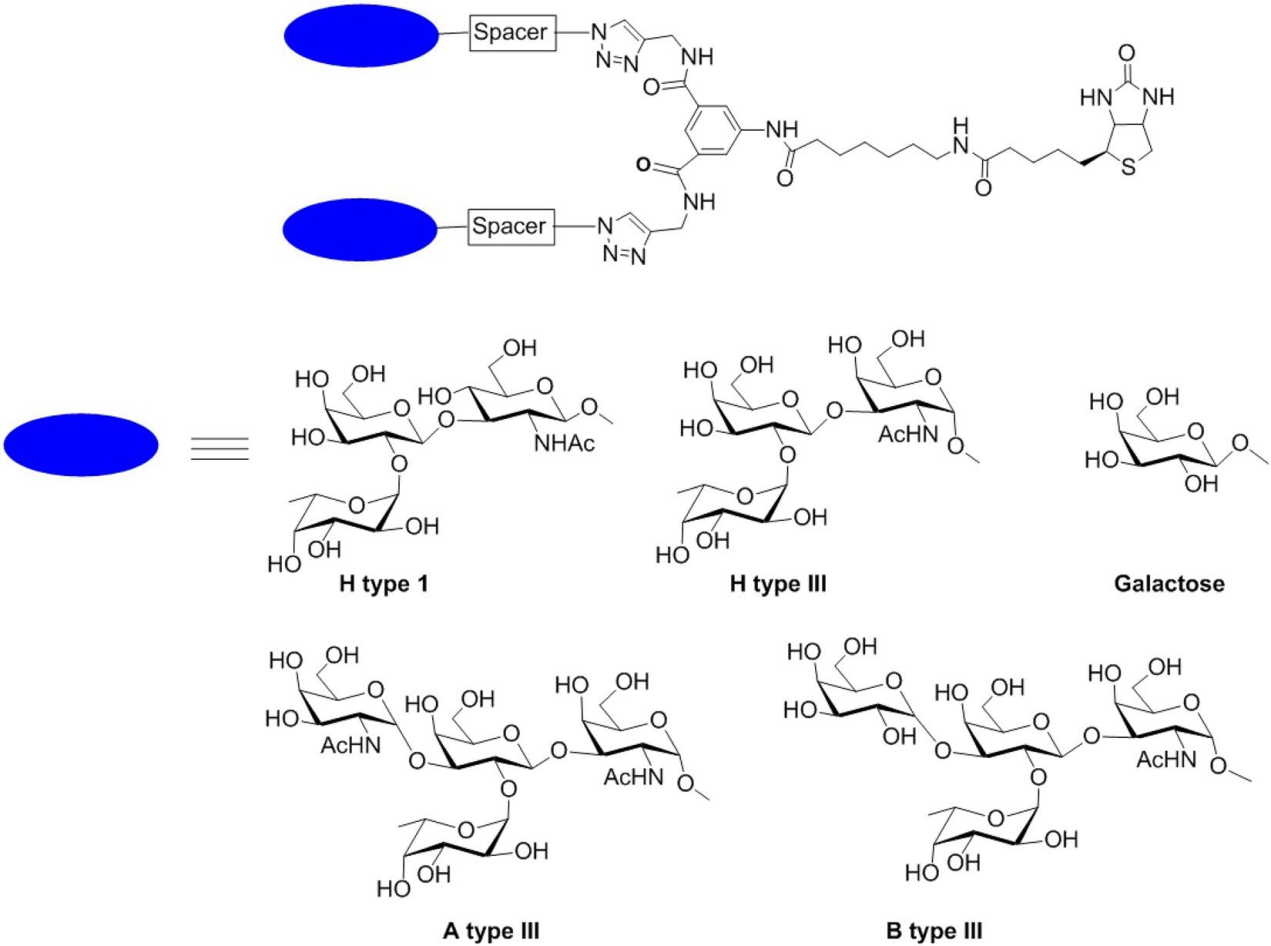
Structures of the bivalent biotinylated glycoconjugates. The blue ellipse represents the glycan headgroup and the spacer is a six-carbon spacer, except for the control galactose AD6, which has a triethylene glycol spacer.

The structures of the compounds used in this study are shown in Fig. 1. The synthesis of the H type I and III have been reported previously, and the synthesis of A and B will be reported elsewhere^9^. To assess the contribution of matrix and subject effects on the binding of SMV to HBGA ligands, bead-binding assays were conducted using a well-described stool and emesis collection from a previous SMV human challenge study^10^. In contrast to stool specimens from outbreaks, this collection allowed the systematic evaluation of the effects of viral titer, stool consistency, and subject-to-subject variability without the risk of bias from variation in exposure or infecting virus strain. Additionally, a pre-challenge stool specimen from each study subject was also tested, which allowed the evaluation of potential false positive recovery.

**Figure 2:**
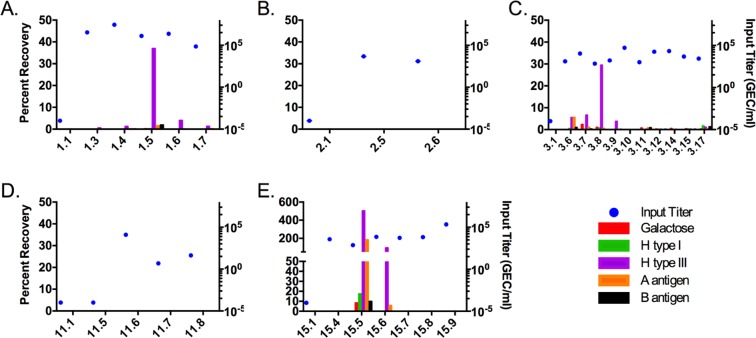
SMV recovery by synthetic HBGA-coated beads varies by subject. Magnetic beads were coated with one of five biotinylated synthetic carbohydrates: H type I, H type III, A antigen, B antigen, or galactose. Percent recovery was adjusted for non-specific binding, assessed with PEG-coated beads. Each panel displays the recovery from stool samples from a single SMV-infected subject. The first sample for each subject (i.e. X.1 sample) is a pre-challenge stool. Blue dots represent the input virus titer for each binding assay. Bars display the adjusted percent recovery from representative experiments. *Sample 11.5 did not generate a virus input titer in the bead binding experiment. The previously determined virus titer for this specimen was plotted as the virus input titer.

A total of 27 SMV-positive stool samples plus five pre-challenge stool samples from five infected challenge subjects were analyzed for virus recovery on magnetic beads coated with biotinylated carbohydrates. Four HBGAs—H type I, H type III, A antigen, and B antigen—have previously been reported to bind noroviruses^5^. Biotinylated galactose was included as a negative control carbohydrate. Biotinylated PEG was used to evaluate non-specific virus binding, and this value was used to adjust the recovery of target carbohydrates. Specific recovery was defined as a PEG-adjusted recovery that was at least three times greater than the PEG-adjusted recovery of galactose-coated beads. Figure 2 shows the recovery of the HBGA-coated beads by subject. Subjects 1, 3, and 15 had one or more stool samples with substantial recovery of SMV on HBGA-coated beads (Fig. 2, panels A, C, and E, respectively). A total of 15 specimens met the definition for specific binding to at least one HBGA, with 4 specimens meeting that criterion for all 4 ligands. In 13 of these specimens, H type III-coated beads specifically bound SMV, compared to 6, 10, and 10 specimens for beads coated with H type I, A antigen, and B antigen, respectively. H type III was the most efficient ligand for SMV recovery, as measured by number of samples with specific recovery and the PEG-adjusted percent recovery. Of the 13 specimens with specific binding to H type III ligands, 9 specimens had at least five-fold greater recovery by H type III-coated beads than beads coated with the other three ligands. However, the PEG-adjusted percent virus recovery by H type III varied widely from 0.00 to 501.91%. Samples 3.6, 15.5, and 15.6 (panels C and E) are the exceptions and showed specific recovery by A antigen-coated beads in addition to H type III-coated beads. Except for Subject 1, each of the subjects had at least one RT-qPCR- positive stool sample with no virus recovery from the ligand-coated beads despite having more than 10^3^ genome equivalent copies (GEC) of SMV in the assay input. In contrast, all three stool samples from Subject 2 did not show any virus recovery with all ligands, despite having high virus input titers (Fig. 2B). Non-specific binding to PEG-coated beads also varied considerably between samples (unadjusted PEG recovery range: 0.00–57.37%) (Table S1). All five pre-challenge samples had no virus recovered on any ligand. These trends were consistent with repeated assays, though the absolute values varied (Figure S1).

**Figure 3:**
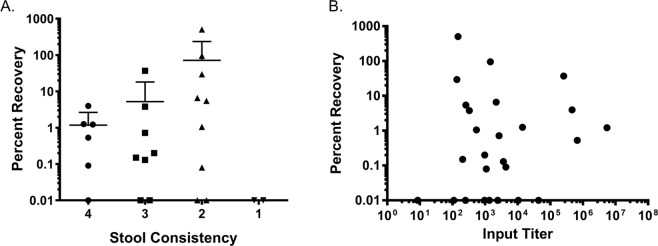
SMV recovery is weakly correlated with stool consistency, but not with input titer. Adjusted percent recovery by H type III-coated beads from SMV-positive stool samples (N = 27) from five challenge subjects is plotted against stool consistency (**A**) and input titer (**B**). Stool consistency was graded by the sample processing technician on the following scale: 4- formed, hard, lumpy; 3- formed, soft, smooth; 2- loose, takes the shape of the container; 1- watery. Two samples did not have stool consistency recorded. Samples with no virus recovery are plotted as 0.01% for visibility.

All the subjects in the challenge study had symptomatic infections, and, the availability of SMV-positive stool samples from the outpatient follow up period allowed evaluation of the impact of stool consistency on virus recovery. Stool consistency was assessed by the sample processing technician at the time of collection using a 4-point scale, where grades 1 and 2 were watery and loose stools, respectively^10^. Generally, virus recovery by H type III-coated beads was greater as the stool consistency was looser (Fig. 3A). However, no virus was recovered from the two grade 1 stools (samples 15.8 and 15.9), despite input virus titers greater than 10^3^ GEC. There was no statistically significant difference in virus recovery by stool consistency (ANOVA, p = 0.6567). Virus input titer (as quantified by RT-qPCR) did not correlate with virus recovery (Pearson’s r = −0.05928, Fig. 3B).

**Figure 4:**
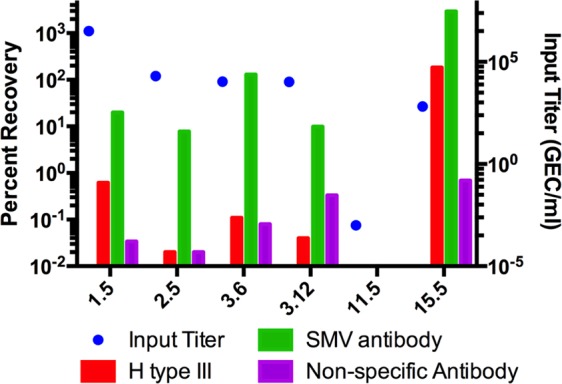
Antibody-coated beads recover more SMV than HBGA-coated beads. Representative SMV-positive stool samples were used to compare recovery of H type III-coated beads and anti-SMV IgG-coated beads. All recoveries were adjusted for non-specific binding using PEG-coated beads. Non-specific IgG was used as a negative control (NC) antibody. Blue dots represent the input virus titer for each binding assay. Bars display the adjusted percent recovery from representative experiments.

The generally low SMV recovery on the HBGA-coated beads could be an indication that only a proportion of the SMV in the samples was capable of binding to the HBGAs or that the available binding sites on the beads became saturated. Two experiments were conducted to explore these hypotheses. The unbound virus in the supernatant from the binding incubation with H type III-coated beads was used for a second round of binding to fresh H type III-coated beads. Two high-titer samples were evaluated (Samples 3.6 and 3.8). In both cases, similar amounts of virus were recovered from the second round of binding as from the first round of binding (Figure S2), indicating that the beads became saturated prior to binding all recoverable virus in the sample. The second experiment compared virus recovery by HBGA-coated beads to recovery by anti-SMV IgG-coated beads (Fig. 4). The antibody-coated beads recovered 16- to 1177-times more virus than H type III-coated beads, suggesting that the poor recovery is specific to the carbohydrate ligands and not the bead-binding assay format.

Only one subject (Subject 2) experienced concomitant vomiting and diarrhea as a result of SMV challenge, allowing a comparison of virus recovery from the two sample types during acute illness. Four emesis samples from this subject had detectable SMV by RT-qPCR, though the virus titers were low (~10^3^ GEC/mL)^13^. Virus recovery from these four emesis samples was evaluated for binding to the synthetic carbohydrates. No binding was detected for any HBGA that was greater than 3 times the non-specific binding to the PEG-coated beads. Non-specific virus recovery from PEG-coated beads for the emesis samples ranged from 0.0–6.9% (Table S2).

## Discussion

Development of HBGA-based norovirus detection assays has been hindered by a lack of assay reliability which could not be explained. In a previous study, examination of stool specimens collected from norovirus outbreaks that had similar titers of the same virus strain yielded dramatically different results when tested by HBGA binding assays^9^. To better understand this variability, stool samples from an SMV human challenge study were used to assess matrix and subject effects on virus recovery by synthetic HBGAs. As seen previously, SMV bound specifically to H type III carbohydrates^5^, but virus recovery varied greatly within and between subjects (Fig. 2). Most subjects had at least one stool sample from which virus could not be recovered despite high input titers, and, for one subject, virus was not recovered from any samples (Fig. 2B), which may indicate a subject-specific effect. Stool consistency was weakly correlated with virus recovery, though the effect was not statistically significant (Fig. 3A). Virus was more likely to be recovered from loose stools than from formed stools, though grade 1 watery stools appeared to inhibit recovery of virus. There was no correlation between input virus titer and virus recovery (Fig. 3B). Taken together, these results suggest that matrix and subject effects strongly influence virus recovery on HBGA-coated beads, emphasizing the need for well-characterized specimen panels for development and validation of candidate diagnostic assay approaches.

The variable recovery of SMV by HBGA ligands could be an artifact of the bead binding assay or it could be due to the biology of the virus. These assays were completed with a single lot of synthetic carbohydrate ligands, thus changes in the ligands is unlikely to be the source of the assay variability. The norovirus capsid protein VP1 interacts with HBGAs through a dimer of the protruding (P) region^14^. Koromyslova *et al*. reported that the P dimer from GII.10 noroviruses binds up to four carbohydrate moieties in a step-wise and dose-dependent manner^15^. If this binding stoichiometry is also true for SMV, then each virus particle could be binding four H type III ligands, reducing the effective binding site molarity by 4-fold. In contrast, each anti-SMV IgG molecule can bind two virus particles. This difference in binding site stoichiometry could explain the low recovery of H type III-coated beads relative to antibody coated beads (Fig. 4).

There was much higher virus recovery from stool samples from Subject 15 compared to those from other subjects, particularly for sample 15.5, which had a PEG-adjusted recovery of 501.91% (Fig. 2E) for H type III. High levels of PCR inhibitors in the stool matrix may have resulted in an underestimate of the virus input titer, which could lead to recoveries greater than 100%. When a column-based RNA extraction method was used with RT-qPCR to quantify the virus input titer, the titer was approximately 2 logs higher than the titer using our standard heat release RNA extraction protocol followed by RT-qPCR (Table S3), supporting the hypothesis that PCR inhibitors may have led to an underestimate of the virus input titer.

There is a wealth of epidemiologic data suggesting that exposure to vomiting is a risk factor for transmission of norovirus. If norovirus can be transmitted through emesis and HBGAs are the initial receptor for the virus, then the virus present in emesis should be recoverable on HBGA-coated beads. However, we could not demonstrate recovery of SMV from RT-qPCR-positive emesis specimens using H type III-coated beads. This failure to recover SMV could be due to subject-specific effects (SMV was also not recovered from stool samples from this subject), lower virus input titers, or matrix effects, such as low pH. There were no SMV-positive emesis samples from other subjects that could be used to address these hypotheses.

## Supplementary information

Supplementary information 
