# Supplementary material for: Snow Mountain Virus recovery by synthetic human histo-blood group antigens is heavily influenced by matrix effects
Source: Sci Rep. 2020 Mar 13;10:4661. doi: 10.1038/s41598-020-60639-6 (PMC7069939; doi:10.1038/s41598-020-60639-6)
Supplement: Supplementary file 1 — Supplementary information [file 41598_2020_60639_MOESM1_ESM.docx]

**Supplementary Material**

**Snow Mountain Virus recovery by synthetic human histo-blood group antigens is heavily influenced by matrix effects**

Amy E. Kirby^1#^*, Yvonne Kienast^1^, Milagros Aldeco^1^, Molly Steele^1^, Abasaheb N. Dhawane^2^, Dandan Liu^2^, Xikai Cui^2^, Amrita Das^2^, Suri Iyer^2, *^, and Christine L. Moe^1^

^1^Center for Global Safe Water, Sanitation and Hygiene, Rollins School of Public Health, Emory University, Atlanta, Georgia, United States of America

^2^Center for Diagnostics and Therapeutics, Department of Chemistry, Georgia State University, Atlanta, Georgia, United States of America

**Table S1. Snow Mountain Virus Recovery on HBGA-Coated Beads**

| **Stool Sample** | **Input Titer^a^ (GEC)** | **Non-Specific Recovery on PEG^b^ (%)** | **PEG-Adjusted Recovery (%)^c^** | | | | |
| --- | --- | --- | --- | --- | --- | --- | --- |
|  |  |  | **Galactose** | **H Type I** | **H Type III** | **A Antigen** | **B Antigen** |
| 1.1 | ND | 0.00 | 0.00 | 0.00 | 0.00 | 0.00 | 0.00 |
| 1.3 | 6.74e+005 | 0.03 | 0.00 | 0.00 | 0.53 | 0.01 | 0.02 |
| 1.4 | 5.51e+006 | 0.01 | 0.00 | 0.04 | 1.22 | 0.16 | 0.07 |
| 1.5 | 2.58e+005 | 0.05 | 0.16 | 0.13 | 36.92 | 1.49 | 1.88 |
| 1.6 | 4.67e+005 | 0.02 | 0.00 | 0.00 | 3.97 | 0.08 | 0.02 |
| 1.7 | 1.41e+004 | 0.07 | 0.00 | 0.01 | 1.25 | 0.16 | 0.04 |
| 2.1 | ND | 0.00 | 0.00 | 0.00 | 0.00 | 0.00 | 0.00 |
| 2.5 | 9.61e+002 | 0.00 | 0.00 | 0.00 | 0.00 | 0.00 | 0.00 |
| 2.6 | 2.49e+002 | 0.00 | 0.04 | 0.00 | 0.00 | 0.00 | 0.00 |
| 3.1 | ND | 0.00 | 0.00 | 0.00 | 0.00 | 0.00 | 0.00 |
| 3.6 | 2.58e+002 | 1.98 | 0.00 | 0.43 | 5.46 | 5.55 | 0.93 |
| 3.7 | 2.18e+003 | 0.22 | 2.35 | 0.1 | 6.6 | 1.04 | 0.19 |
| 3.8 | 1.36e+002 | 1.83 | 1.02 | 0.66 | 29.46 | 0.54 | 0.03 |
| 3.9 | 3.29e+002 | 0.22 | 0.01 | 0.07 | 3.78 | 0.07 | 0.06 |
| 3.10 | 1.05e+004 | 0.00 | 0.00 | 0.00 | 0.00 | 0.00 | 0.00 |
| 3.11 | 2.06e+002 | 1.56 | 0.67 | 0.00 | 0.15 | 0.78 | 0.82 |
| 3.12 | 3.64e+003 | 0.49 | 0.22 | 0.11 | 0.13 | 0.04 | 0.12 |
| 3.14 | 4.35e+003 | 0.95 | 0.49 | -0.02 | 0.09 | 0.01 | -0.01 |
| 3.15 | 9.73e+002 | 0.27 | 0.36 | 0.24 | 0.2 | 0.08 | 0.11 |
| 3.17 | 5.43e+002 | 2.34 | 0.27 | 1.65 | 1.05 | 0.35 | 1.21 |
| 11.1 | ND | 0.00 | 0.00 | 0.00 | 0.00 | 0.00 | 0.00 |
| 11.5 | 1.1e+002 | 29.91 | 0.00 | 0.00 | 0.00 | 0.00 | 0.00 |
| 11.6 | 2.46e+003 | 0.00 | 0.00 | 0.00 | 0.00 | 0.00 | 0.00 |
| 11.7 | 9.62e-001 | 0.00 | 0.00 | 0.00 | 0.00 | 0.00 | 0.00 |
| 11.8 | 8.59e+000 | 22.94 | 0.00 | 0.00 | 0.00 | 0.00 | 0.00 |
| 15.1 | ND | 0.00 | 0.00 | 0.00 | 0.00 | 0.00 | 0.00 |
| 15.4 | 2.68e+003 | 0.00 | 0.00 | 0.00 | 0.72 | 0.00 | 0.00 |
| 15.5 | 1.48e+002 | 50.22 | 8.31 | 17.37 | 501.91 | 182.96 | 9.8 |
| 15.6 | 1.45e+003 | 1.53 | -5 | -4.59 | 94.92 | 5.68 | -2.42 |
| 15.7 | 1.09e+003 | 1.99 | -0.23 | -0.25 | 0.08 | -0.01 | -0.13 |
| 15.8 | 1.35e+003 | 57.37 | -14.65 | -31.22 | -1.22 | -25.84 | -8.17 |
| 15.9 | 4.4e+004 | 0.19 | -0.03 | -0.03 | -0.02 | -0.03 | -0.03 |

**^a^**Number of virus genomes in assay based on virus titer (GEC/ml) of stool sample

^b^Percent recovery on PEG-coated beads without any HBGA carbohydrates

^c^Percent recovery on HBGA-coated beads minus the non-specific recovery on PEG-coated beads.

**Table S2. Snow Mountain Virus Recovery from Emesis Samples**

| **Emesis Sample** | **Input Titer^a^ (GEC)** | **Non-Specific Recovery on PEG^b^ (%)** | **PEG-Adjusted Recovery (%)^c^** | | | | |
| --- | --- | --- | --- | --- | --- | --- | --- |
|  |  |  | **Galactose** | **H Type I** | **H Type III** | **A Antigen** | **B Antigen** |
| 2.1 | 5.28 | 0.0 | 0.0 | 0.0 | 0.0 | 0.0 | 0.0 |
| 2.2 | 613 | 2.1 | -1.1 | -1.6 | -0.4 | 1.6 | -0.7 |
| 2.3 | 563 | 6.9 | -6.9 | -6.9 | -6.9 | -5.5 | -4.6 |
| 2.4 | 590 | 2.5 | -0.9 | -2.0 | -1.4 | -2.5 | -2.4 |

**^a^**Number of virus genomes in assay based on virus titer (GEC/ml) of emesis sample

^b^Percent recovery on PEG-coated beads without any HBGA carbohydrates

^c^Percent recovery on HBGA-coated beads minus the non-specific recovery on PEG-coated beads.

**Table S3. Comparison of Heat Release to Commercial RNA Isolation Kit for SMV Virus Recovery on H Type III-Coated Beads**

| **Sample** | **Method^a^** | **Input (Ct)** | **PEG (Ct)** | **H Type III (Ct)** |
| --- | --- | --- | --- | --- |
| 1.3 | Heat Release | No Ct | 34.0 | 28.5 |
| 1.3 | Commercial Kit | 21.2 | 33.3 | 34.1 |
| 11.8 | Heat Release | 37.8 | No Ct | No Ct |
| 11.8 | Commercial Kit | 37.4 | 39.7 | No Ct |
| 15.5 | Heat Release | 31.5 | 38.7 | 32.9 |
| 15.5 | Commercial Kit | 30.3 | 36.1 | No Ct |

**^a^**Heat release as described in the methods section. Commercial kit indicates samples that were prepared using the Qiagen QiaAmp Viral RNA Mini kit per manufacturer’s instructions.


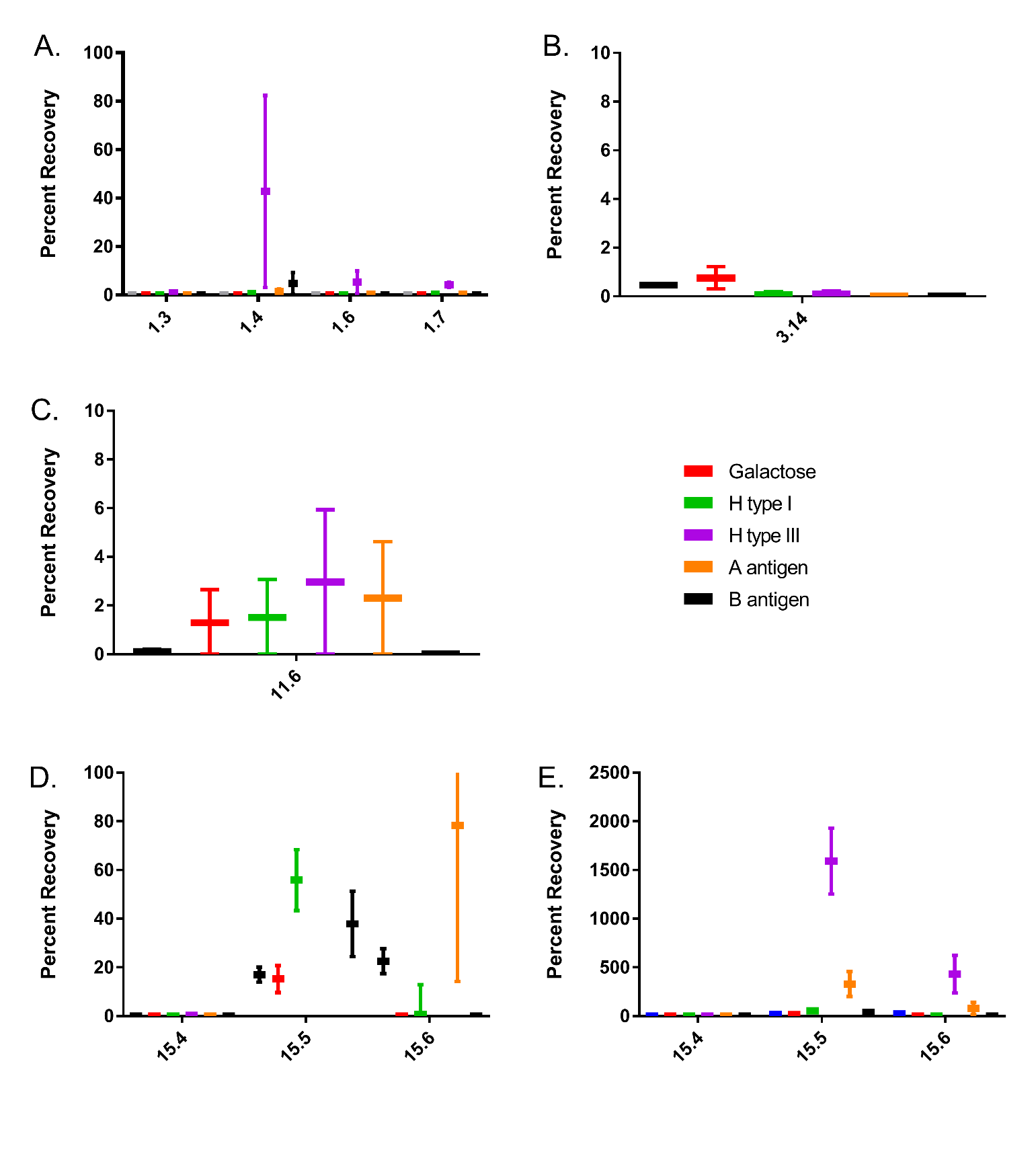


Figure S1. Assay replicates on selected SMV-positive samples. Nine stool samples were selected for repeated assays because they had high virus titers but no or minimal specific recovery { Figure S1A, (samples 1.3, 1.4, 1.6, 1.7), Figure S1B (sample 3.14) and Figure S1C (sample 11.6) or very high specific recovery {Figures S1D or E, (samples 1.5, 15.5, 15.6). The range of the repeated measures is plotted for each sample and each HBGA ligand. Panels D and E use the same data but with different y-axis scales for visualization.


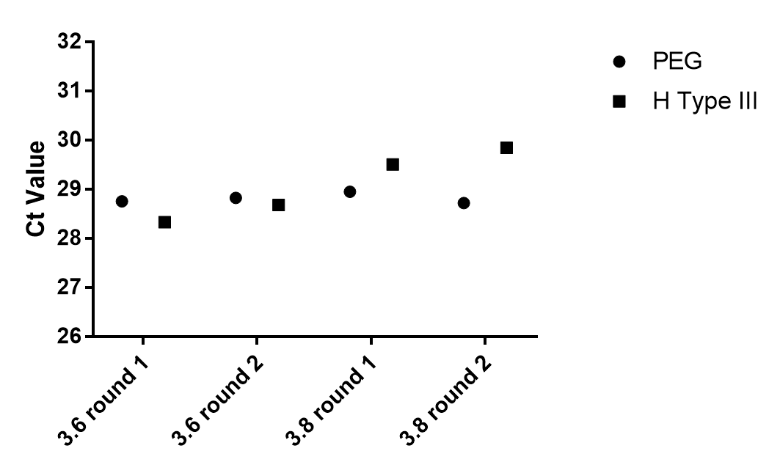


Figure S2. Additional SMV can be recovered from the unbound fraction of H type III-coated beads. For two high titer samples, the supernatant after sample incubation with the H type III-coated beads (round 1) was recovered and used as the input for a second assay with fresh H Type III-coated beads (round 2). Ct values from the SMV-specific qPCR are reported.
